# Attitudes of Spanish hospital staff towards COVID-19 vaccination and vaccination rates

**DOI:** 10.1371/journal.pone.0257002

**Published:** 2021-09-10

**Authors:** Guillermo Mena, Beatriz Blanco, Irma Casas, Antonia Huertas, María-Araceli Sánchez, Mario Auñón, Jordi Viñas, María Esteve

**Affiliations:** 1 Preventive Medicine Department—Germans Trias i Pujol University Hospital, Badalona, Spain; 2 Autonomous University of Barcelona, Badalona, Spain; 3 Germans Trias I Pujol Research Institute (IGTP), Badalona, Spain; National Center for Global Health and Medicine, JAPAN

## Abstract

**Background:**

COVID-19 vaccine hesitancy seems to be universal across countries and subgroups, and so are its determinants. We studied the willingness and factors associated with the decision to be vaccinated against COVID-19 in healthcare workers (HCW) in a Spanish tertiary hospital. Furthermore, we compared the percentage of willingness to vaccinate against COVID with actual vaccination rates among HCW in our hospital.

**Methods:**

From December 21, 2020 to January 4, 2021, before initiation of the COVID-19 HCW vaccination campaign at Germans Trias i Pujol University Hospital (HUGTiP), an anonymous self-administered questionnaire was administered to HCW. Univariate and multivariate logistic regression of the association of variables with the outcome “intention to receive the COVID-19 vaccine as soon as possible” was conducted. Vaccination rates were extracted from the hospital information systems.

**Results:**

Forty-four percent of HCW included in the study declared a willingness to be vaccinated against COVID-19 as soon as possible. This was associated with male sex [1.66 (95%CI 1.13–2.43); p = 0.009], older age [1.02 (95%CI 1.00–1.03); p = 0.014], belonging to the occupational groups “physician” or “other” [5.76 (95%CI 3.44–9.63) and 2.15 (95%CI 1.25–3.70); p<0.001], respectively, and reporting influenza vaccination during the last three seasons or at least one of the last three seasons [3.84 (95%CI 2.56–5.75) and 2.49 (95%CI 1.71–3.63); p<0.001]. One in ten hospital workers reported they were unwilling to receive COVID-19 vaccination. Actual COVID-19 vaccination uptake among HCW was higher (80.4%) than the percentage of willingness to vaccinate estimated from the questionnaire. Physicians not only had the highest vaccination rate, but also the highest correlation between the reported intention to vaccinate and the final decision to receive COVID-19 vaccination.

**Conclusions:**

COVID-19 vaccination uptake was higher than previously estimated according to the stated intentions of HCW. Doubts and fears must be addressed, particularly in persons less inclined to be vaccinated: females, younger people and those not vaccinated against influenza in recent seasons. The study of barriers and strategies aimed at promoting COVID-19 vaccination must be adapted in relation to occupational groups’ attitudes, understanding their idiosyncrasies with respect to this and other vaccines.

## Introduction

As of January, 2021 (week 2021–1), 89,802,096 cases of COVID-19 have been reported worldwide, including 1,940,529 deaths. Europe accounted for 28,291,217 cases; the five countries reporting the most cases were Russia (3,425,269), the United Kingdom (3,072,349), France (2,783,256), Italy (2,276,491) and Spain (2,111,782), with Spain reporting 52,275 deaths [1].

On December 21, 2020, the European Medicines Agency (EMA) recommended granting conditional marketing authorization for the Pfizer/BioNTech COVID-19 vaccine in people aged ≥ 16 years in the EU. At the time of writing, the EMA Committee for Medicinal Products for Human Use was carrying out a rolling review of three vaccines (AstraZeneca/Oxford, Janssen-Cilag International NV, and Moderna). Moderna had also applied to the EMA for conditional marketing authorization [2].

The prioritization of COVID-19 vaccination should take into account various dimensions and requires contextualization. Although vaccinating adults aged 18–59 years is not the most effective or efficient strategy when vaccine supply is limited, consideration should be given to specific groups or settings that may have a higher risk of exposure. The vaccination of healthcare workers is beneficial, as it improves the resilience of the healthcare system. The benefit would be greater if the vaccine were effective against infection and, therefore transmission, since it would offer indirect protection to patients, residents of long-term care facilities and other high-risk individuals [3].

On December 27, 2020, Spain started administering the Pfizer-BioNTech vaccine to residents and health and social health workers in nursing homes for the elderly and disabled. On January 4, 2021, vaccination of all front-line healthcare workers began in Catalonia [4].

Covid-19 vaccine hesitancy seems to be universal across countries, states, and subgroups (including healthcare providers and parents), and so are its determinants—perceived disease or outbreak severity, infection risk, and vaccine safety, effectiveness, and necessity [5]. Nevertheless, differences in acceptance rates range from almost 90% in China to < 55% in Russia. The rate of vaccine acceptance in Spain is 74% [6].

The objective of this study was to evaluate the acceptance of COVID-19 vaccination in HCW (care and non-care staff) in a Spanish tertiary hospital. Sociodemographic and clinical variables associated with the decision to be vaccinated were analyzed. We compared the percentage of willingness to vaccinate against COVID-19 with actual vaccination rates among HCW in our hospital.

## Material and methods

A cross-sectional study was conducted among HCW of the Germans Trias i Pujol Hospital (HUGTiP), a public university hospital with 700 beds. HCW were surveyed from December 21, 2020 to January 4 2021, before the COVID-19 (HCW) vaccination campaign started at HUGTiP. The aim was to recruit the maximum number of subjects, and therefore the entire HUGTiP staff (n = 4,402) were invited to participate.

On January 4, 2021, the COVID-19 vaccination campaign for HUGTiP staff began. February 25 was the last call for the first vaccination dose during this specific campaign. Vaccination rates were calculated among HCW offered the first dose of COVID-19 vaccine during this period.

### Questionnaire

We used an anonymous self-administered questionnaire created purposely for this study (S1 Appendix). The questionnaire was piloted in ten HCW in advance of the main study, to ensure it addressed the principal study aims and was comprehensible. Completing the full questionnaire took about six minutes. The written questionnaire was distributed to all HCW. Study researchers visited all hospital services and departments on three occasions, inviting all HCW they met to participate. Researchers explained the aim of the study and the anonymous character of the questionnaire. Although the questionnaire was self-administered, researchers were on hand to answer any doubts. The surveys were collected on the spot.

### COVID-19 vaccination rates

After the first dose of Covid-19 vaccine was administered (February 25), vaccination rates were extracted from hospital information systems, anonymized, and aggregated by sex and occupational category.

### Study variables

Sociodemographic data collected included age (Q1), sex (Q2) and occupation (Q3). Participants were asked about their daily contact with patients (Q4). In terms of clinical data, participants were asked if they belonged to any influenza risk group (Q9) and whether they had been vaccinated during the current influenza season (2020–2021) (Q5) and/or during any of the previous two seasons (2019–2020 and 2018–2019) (Q6 & Q7). Finally, they were asked about their intention to be vaccinated against COVID-19 (Q8).

Secondary variables were created by extracting from the answers: *Occupational group* from the answer to Q3 (“Physicians”, “Nurses”, “Nursing assistants”, “Porters”, “Administrative staff” and “Other”); *Full adherence with seasonal influenza vaccination* (Option “yes” in Q5 plus Q6 plus Q7); *Intermediate adherence to seasonal influenza vaccination* (Option “yes” in Q5 plus Q6, or Q6 plus Q7, or Q5 plus Q7); *Underlying health conditions* (Any option in Q9); *Intention to receive the COVID-19 vaccine as soon as possible* (option “A” in Q8), *Intention to receive the COVID-19 vaccine* (options “A” or “B” in Q8); *Hesitation about receiving the COVID-19 vaccine* (option “C” in Q8); *Declining COVID-19 vaccination* (options “D” or “E” in Q8).

### Statistical analysis

Since the primary endpoint was to assess the intention to be vaccinated against COVID-19, the statistical analysis was made using questionnaires in which Q8 was completed correctly. Questionnaires with missing data for the variable of interest were not included in the respective analyses. The Kolmogorov-Smirnov test was performed to determine the normality of the variable “age”. The results are presented as frequencies and percentages for categorical variables and, according to distribution, as means and standard deviation (SD) or median and interquartile range (IQR). The Chi-square and Mann-Whitney U tests were used to compare groups. A univariate analysis for the association of each variable with the outcome variable “*Intention to receive the COVID-19 vaccine as soon as possible*” was initially carried out. Adjusted odds ratios and 95% confidence intervals (CI) were estimated. Variables with a p value < 0.1 in the univariate analysis were candidates for inclusion in the multivariate logistic regression analysis using a stepwise approach. Only variables showing good evidence of association (p value < 0.05) with the outcome variable were retained in the final model. Data were managed in Microsoft Excel and analyses were performed using IBM SPSS Statistics for Windows, version 24 (IBM Corp., Armonk, N.Y., USA).

### Ethical aspects

The planning, carrying out and reporting of the study were in line with the Declaration of Helsinki World Medical Association [7]. An anonymous self-administered questionnaire was used. We obtained verbal consent from all study participants. The research protocol was approved by the HUGTiP Ethics Committee (ref: PERCEPFLU_20_21). No incentives were provided for participation.

## Results

Of the 923 completed questionnaires, 906 (98.2%) were considered valid, giving a participation rate of 20.6% of the overall workforce. Considering only valid information per variable: 75.2% were female, 23.3% male and 4.5% did not state their sex. The median age was 34 years (IQ 26–47). The age range was 18–66 years, with 4.8% aged ≥ 60 years, and the age was unknown in 5.1%. Respondents were categorized into six main groups by occupation: Nurses (25.8%), physicians (22.3%), auxiliary nurses (15.5%), porters (4.6%), administrative staff (9.4%) and others (17%). In 49 subjects (5.4%) the occupation was missing. In terms of professional contact with patients, the survey reported “very close” (65.5%), “close or medium” (13.1%) and “minimum or no contact” (21.3%). Only one respondent did not declare their contact with patients. Among respondents, the most frequently reported health conditions were respiratory disease (n = 54), cardiovascular disease (n = 31), diabetes mellitus (n = 19), inflammatory disease (n = 18), immunosuppression (n = 13), obesity (n = 10), celiac disease (n = 10), cancer (n = 6), renal disease (n = 5), neurological disease (n = 5), salicylate intolerance (n = 5), liver disease (n = 2) and neuromuscular disease (n = 1). Ten women reported they were pregnant. Overall, 17.7% and 5.3% of respondents reported having ≥1 or ≥2 health conditions, respectively.

Table 1 shows the differences between responders and the total of hospital workers with respect to sex and occupational category. The percentage of nurses was significantly lower in responders (27.3% vs. 31.8%; p<0.01). The proportion of female responders was non-significantly higher in respondents than all workers (75.6% vs. 72.9%).

**Table 1:** Description of hospital workers included in the study vs. overall population.

Characteristics	Overall	Study population	p
	(n = 4,402)*	(n = 906)^#^	
**Female, n (%)**	72.9%	75.6%	0.09
**Occupational group, n (%)** ^**¬**^			
Physician	25.1%	23.6%	0.34
Nurse	31.8%	27.3%	**<0.01**
Auxiliary nurse	17.8%	16.3%	0.3
Porter	6.1%	4.9%	0.16
Administrative staff	9.3%	9.9%	0.55

*In 80 subjects, sex was missing from the records.

^#^In 41 subjects, sex was missing in the questionnaire; In 49 subjects, occupational group was missing in the questionnaire.

¬Individuals working in other occupational groups are not represented in this table.

Forty-four percent of HCW included declared a willingness to be vaccinated against COVID-19 as soon as possible. The questionnaire results according to demographic, occupational, and clinical characteristics are summarized in Table 2. More males had decided to be vaccinated, and more females stated "I don’t know yet". Likewise, the proportion of doctors who had decided to be vaccinated was clearly higher than the other categories. The categories with the highest level of indecision were administrative staff and nursing assistants. Nursing assistants were also more likely to reject vaccination, followed by porters. The proportion of respondents not receiving the influenza vaccination during the current season and not having received the influenza vaccination during the last three seasons was distributed similarly in the professional categories. Participants who had received the influenza vaccine intermittently tended to accept the COVID-19 vaccine (marking options 1 or 2 in Q8). There were no significant differences with respect to contact with patients in the decision to be vaccinated against COVID-19 (p = 0.56). Having ≥ 1 health condition was not associated with a greater propensity to be vaccinated in respondents (p = 0.45). The univariate analysis (Table 3) showed that “willingness to vaccinate as soon as possible” was associated with male sex [1.56 (95%CI 1.17–2.18); p = 0.003], older age [1.02 (95%CI 1.01–1.03); p = 0.003], belonging to the occupational groups “physician”, “nurse” or “other” [8.22 (95%CI 5.09–13.27), 1.72 (95%CI 1.1–2.69), and 2.08 (95%CI 1.27–3.70); p<0.001] respectively, and reporting influenza vaccination during the last three seasons or at least one of the last three seasons [5.19 (95%CI 3.62–7.46) and 2.56 (95%CI 1.82–3.6); p<0.001]. The multivariate logistic regression model (Table 3) showed that “willingness to vaccinate as soon as possible” was associated with male sex (1.66 (95%CI 1.13–2.43); P = 0.009], older age [1.02 (95%CI 1.00–1.03); p = 0.014], occupational groups “physician” or “other” [5.76 (95%CI 3.44–9.63) and 2.15 (95%CI 1.25–3.70); p<0.001], respectively, and reporting influenza vaccination during the last three seasons or at least one of the last three seasons [3.84 (95% CI 2.56–5.75) and 2.49 (95%CI 1.71–3.63); p<0.001].

**Table 2:** Attitudes towards receiving the COVID-19 vaccine by demographic, occupational, and clinical characteristics.

Characteristics	n	Yes, as soon as possible	Yes, but I will wait a few weeks/months	I don´t know yet	I don´t think so	Absolutely not	p
**Sex, n (%)**							0.017
Female	654	40.5	17.6	30.6	8.0	3.4	
Male	211	52.1	17.1	19.4	8.1	3.3	
**Age, median (IQR)**	860	36 (28–49)	30.5 (25–47)	35 (26–46)	32 (25–45.5)	29(25–37)	0.02
**Occupational group, n (%)**							<0.001
Physician	202	74.3	11.4	13.4	0.5	0.5	
Nurse	234	37.6	20.1	29.5	10.7	2.1	
Auxiliary nurse	154	26.0	19.5	35.7	12.3	6.5	
Porters	42	33.3	21.4	31.0	9.5	4.8	
Administrative staff	85	31.8	18.8	37.6	8.2	3.5	
Other*	189	42.3	18.5	27.0	7.4	4.8	
**Contact with patients, n (%)**							0.563
Very close	593	43.3	18.0	27.0	7.9	3.7	
Close or medium	119	52.1	13.4	27.7	5.9	0.8	
Minimum or No contact	193	41.5	19.2	28.0	7.8	3.6	
**Influenza vaccination status during the current season (2020–2021), n (%)**							<0.001
Vaccinated	275	56.7	17.9	21.2	2.5	1.6	
Non-vaccinated	118	28.8	18.6	34.1	14.1	5.4	
**Influenza vaccination status for the last seasons** ^#^							<0.001
Always vaccinated	249	64.3	15.7	17.3	2.0	0.8	
Intermittent vaccinated	298	47.0	20.1	24.5	5.7	2.7	
Never vaccinated	315	25.7	17.8	36.2	14.6	5.7	
**Number of health conditions^**							0.447
None	746	44.5	17.7	26.3	8.3	3.2	
At least one	160	41.9	17.5	31.9	5.0	3.8	

*Other: Researchers, nutritionists, pharmaceutics, physiotherapists, psychologists, social assistants, IT staff, security staff, cleaning workers, engineers, maintenance staff, laboratory, radiology occupational health and pharmacy technicians among others.

#Last three seasons: 2018–2019; 2019–2020; 2020–2021.

^Health conditions: Diabetes mellitus, morbid obesity (BMI >40), chronic renal disease or nephrotic syndrome, cardiovascular disease (including hypertension), neurologic disease, respiratory disease, hemoglobinopathies and/or anemia, hemophilia, other bleeding disorders and chronic bleeding disorders, as well as recipients of blood products and multiple transfusions, asplenia or severe splenic dysfunction, chronic hepatic disease (including chronic alcoholism), neuromuscular disease, immunosuppression [including primary, secondary viral, drug (also Eculizumab) immunodeficiencies, transplant recipients, and complement deficiency], cancer and malignant blood diseases, cochlear implant or pending, cerebrospinal fluid fistula, celiac disease, chronic inflammatory disease, prolonged treatment with acetylsalicylic acid and pregnancy or puerperium (up to 6 months after delivery).

**Table 3:** Univariate and multivariate logistic regression of factors associated with the intention to receive COVID-19 vaccination as soon as possible.

Characteristics	Univariate			Multivariate		
	OR	95% CI	p-value	OR	95% CI	p-value
**Male sex**	1.599	1.170–2.184	0.003	1.662	1.135–2.433	0.009
**Age**	1.017	1.006–1.028	0.003	1.016	1.003–1.029	0.014
**Occupational group**	<0.001			<0.001		
Auxiliary nurse	Ref.	Ref.		Ref.	Ref.	
Administrative staff	1.327	0.742–2.237	0.341	1.428	0.758–2.692	0.270
Porter	1.425	0.683–2.974	0.345	1.161	0.475–2.841	0.743
Other*	2.076	1.269–3.396	0.004	2.152	1.252–3.701	0.006
Nurse	1.718	1.099–2.686	0.018	1.617	0.998–2.620	0.051
Physician	8.221	5.093–13.27	<0.001	5.757	3.441–9.632	<0.001
**Contact with patients**	0.153					
Minimum or No contact	Ref.	Ref.				
Close or medium	1.536	0.970–2.433	0.067			
Very close	1.080	0.777–1.502	0.645			
**Influenza vaccination status for the last seasons** ^#^	<0.001			<0.001		
Never vaccinated	Ref.	Ref.		Ref.	Ref.	
Intermittently vaccinated	2.56	1.822–3.596	<0.001	2.488	1.706–3.630	<0.001
Always vaccinated	5.194	3.616–7.459	<0.001	3.836	2.559–5.752	<0.001
**At least one health condition^**	0.898	0.636–1.269	0.543			

*Other: Researchers, nutritionists, pharmaceutics, physiotherapists, psychologists, social assistants, IT staff, security staff, cleaning workers, engineers, maintenance staff, laboratory, radiology occupational health and pharmacy technicians among others.

#Last three seasons: 2018–2019; 2019–2020; 2020–2021.

^Health conditions: Diabetes mellitus, morbid obesity (BMI >40), chronic renal disease or nephrotic syndrome, cardiovascular disease (including hypertension), neurologic disease, respiratory disease, hemoglobinopathies and/or anemia, hemophilia, other bleeding disorders and chronic bleeding disorders, as well as recipients of blood products and multiple transfusions, asplenia or severe splenic dysfunction, chronic hepatic disease (including chronic alcoholism), neuromuscular disease, immunosuppression [including primary, secondary viral, drug (also Eculizumab) immunodeficiencies, transplant recipients, and complement deficiency], cancer and malignant blood diseases, cochlear implant or pending, cerebrospinal fluid fistula, celiac disease, chronic inflammatory disease, prolonged treatment with acetylsalicylic acid and pregnancy or puerperium (up to 6 months after delivery).

During January-February 2021, the first dose of COVID-19 vaccine was administered to 2541 hospital workers (vaccination rate 80.4%). The comparison between willingness to vaccinate (“vaccine uptake as soon as possible” and “intention to get vaccinated”) and actual vaccine uptake rate by sex and occupational category is summarized in Table 4. Vaccination rates were significantly higher (80.4%) than the percentage of persons declaring their intention to be vaccinated in the survey (80.4 vs. 61.3%, p<0.001). Both females and males showed a higher percentage of vaccination than the percentage of willingness to vaccinate. Among occupational groups, physicians had the highest vaccination rate as predicted by a previous survey. Although the percentage of willingness to vaccinate for nurses, auxiliary nurses, porters, and administrative staff was around 50.6–57.9%, the actual vaccination rates were much higher (72.2–82.1%).

**Table 4:** COVID-19 vaccination rates vs. previous intention to vaccinate.

	Data from hospital information systems		Data from the study questionnaire		
	Overall population (n = 4,402)*	Vaccination rates (80.4%)	Study population(n = 906) ^#^	Vaccination as soon as possible (44%)^a^	Intention to vaccinate (61.3%)^b^
**Sex**					
Female	3149*	81.6%	654^#^	40.5%	58.1%
Male	1173	82.9%	211	52.1%	69.2%
**Occupational group**¬					
Physician	1106	85.2%	202	74.3%	85.6%
Nurse	1401	81.5%	234	37.6%	57.7%
Auxiliary nurse	784	75.5%	140	26.0%	57.9%
Porter	270	72.2%	42	33.3%	54.8%
Administrative staff	408	82.1%	85	31.8%	50.6%

*In 80 subjects, sex was missing from the record.

^#^In 41 subjects, sex was missing in the questionnaire; In 49 subjects, occupational group was missing in the questionnaire.

¬Individuals working in other occupational groups are not represented in this table.

^a^ Intention to receive COVID-19 vaccine as soon as possible (option “A” at Q8).

^b^ Intention to get COVID-19 vaccination (options “A” or “B” at Q8).

## Discussion

Forty-four percent of the HCW included in this study declared a willingness to be vaccinated against COVID-19 as soon as possible. Taking other HCW who were fairly convinced they would receive the vaccination but preferred to wait some time into account, the willingness to be vaccinated extended to almost two thirds of the study population. One in ten hospital workers reported they were unwilling to receive COVID-19 vaccination. Male sex, older age, working as a physician and influenza vaccination history were factors related to the willingness to receive the COVID-19 vaccine as soon as possible.

The willingness of HCW to receive COVID-19 vaccination has been little studied to date. A recent meta-analysis of 8847 HCW meeting the inclusion criteria showed a wide range of results between the eleven studies included. The overall proportion of HCW who intended to accept COVID-19 vaccination was 55.9% (95% CI: 43.6–67.9%) with a range between studies of 27.7%-81.5% [8]. Our results are very similar to those obtained by a survey of 2,678 HCW in France and French-speaking parts of Belgium and Canada, which showed 72.4% of participants would be in favor of being vaccinated against COVID-19 (48.6% with high acceptance and 23% with moderate acceptance) [9].

In general, males are more likely to accept any kind of vaccination [10]. We found no significant differences between the proportions of men and women declining vaccination, but the percentage of men who wanted vaccination as soon as possible was higher than in women (52.1% vs 40.4%). Conversely, the proportion of women who were hesitant was higher than in men (30.6 vs. 19.4%). Older people tend to be more compliant with vaccination guidelines, especially for influenza vaccination. In addition, older healthcare workers usually have a significantly higher uptake of vaccines, in general [11]. Our results show an association between age and high acceptance of COVID-19 vaccination. The age-related severity of SARS-Cov-2 infection may increase the perception of risk in older HCW, as usually happens with the influenza vaccination.

In terms of occupational groups, our results are aligned with the limited current evidence, which shows that physicians are more inclined to receive COVID-19 vaccination than other HCW [8] and that nurses are more prone to receive vaccination than auxiliary nurses and other categories [8, 9]. These results are aligned with those concerning influenza vaccination coverage, which is usually higher in physicians, followed by nurses and then by the other occupational categories [12–14]. The attitudes of occupational groups towards vaccination may be correlated with the educational level. In our study, the professional categories that required a university degree (not only physicians and nurses, but also physiotherapists, pharmacists, and nutritionists, among others) showed a greater intention to be vaccinated as soon as possible. In a recent cross-sectional study in American HCW, vaccine acceptance was significantly associated with increasing age, education, and income [15]. We hypothesize that scientific education could be linked to a better understanding of vaccine mechanisms and greater trust in scientific methods and evidence-based medicine. Furthermore, these professionals are used to searching for scientific information from trusted sources, since they need it for their daily work.

It is known that previous uptake of the seasonal influenza vaccine is positively associated with the intention to vaccinate and receipt of influenza vaccines [16]. In the case of COVID-19 vaccination, a review that compared trends and synthesized findings in vaccination receptivity over time across the US and internationally, concluded that the past vaccination history, including the influenza and the measles, mumps, and rubella (MMR) vaccines, was a proven indicator of vaccine acceptance [17].

Unexpectedly, underlying health conditions and closeness of contact with patients were not associated with the intention to vaccinate. In the previously cited meta-analysis [8], HCW with chronic conditions were more prone to receive COVID-19 vaccination. However, we found no association between any of the reported health conditions, either one by one, or using the categorical variable “at least one health condition”, and a high or moderate willingness to be vaccinated. These results were totally unexpected, as hypertension, followed by diabetes, and cardiovascular disease are the most common comorbidities seen in COVID-19 positive patients consulting health-care settings worldwide [18]. As the prognostic effect of clinical conditions on COVID-19 mortality varies substantially according to the mean age [19], the relationship between health conditions and willingness to vaccinate in subjects aged > 50, > 55 and > 60 years was analyzed, but we found no association. The nature of this variable, containing sensitive information, could lead to underreporting of health conditions. Taking hypertension as an example, one in three adults in Spain is hypertensive (66% in those aged > 60 years) [20]. In addition, the prevalence of masked hypertension in HCW in Spain is almost 25% [21]. In our study, only 3.4% of HCW reported cardiovascular disease (including hypertension).

Individual perceived risk has repeatedly been described as a factor associated with the intention to receive influenza vaccination [22, 23] and the Covid-19 vaccine [24]. Our results show that participants in close or medium contact with patients are more prone to be vaccinated than those with very close or minimum contact with patients, which is difficult to interpret. Combining closeness and frequency in the same question could have led to different interpretations of this question.

During the first two months of 2021, four out of five HUGTiP hospital workers received the first of shot of the COVID-19 vaccine. The vaccination rate was higher than expected from the questionnaire answers (three out of five hospital workers potentially vaccinated). As expected, physicians had the highest vaccination rates (85.2%). All other categories had higher vaccination rates than expected according to the survey results, especially administrative staff and nurses, who had the largest differences between intention and uptake.

HCW are more likely to be compliant or have a positive change in intention to receive influenza vaccine. Although there is disinformation on the influenza vaccine and many HCW have erroneous beliefs about it, this does not seem to affect the change between intention and vaccination [25]. The case of COVID-19 vaccination is different, as the quantity of new daily information received may have changed the decisions of HCW in the time between the survey and vaccination. However, the information on immunogenicity and safety coming from other countries during this period was encouraging, possibly meaning more people were vaccinated than expected by the survey.

To our knowledge, this is the first study to evaluate the intention to vaccinate and COVID-19 vaccination rates of Spanish HCW and to analyze variables such as previous influenza vaccination and individual risk factors in HCW attitudes’ to receiving the COVID-19 vaccine. The main limitation of the study is that it was carried out in a single center, with a participation rate of 20.6%, which may affect the generalization of the results. However, the comparisons between the respondents and the total of workers were made using the only two variables available for all workers: sex and occupational category. Likewise, as the sample was not randomized, nurses were underrepresented. In addition, variables such as “previous COVID disease” or “relatives with underlying conditions” that might have acted as confounding variables were not collected. Finally, as vaccination rates were obtained through aggregated information, we could not compare the intention to vaccinate and the final decision individually.

COVID-19 vaccination uptake was higher than previously estimated according to the stated intentions of HCW. Physicians not only had the highest vaccination rates, but also the highest correlation between the reported intention and the final decision to receive the COVID-19 vaccination. Among unvaccinated workers, doubts and fears must be addressed. The study of barriers and strategies aimed at promoting COVID-19 vaccination must be adapted in relation to occupational groups’ attitudes, understanding their idiosyncrasies with respect to this and other vaccines.

## Supporting information

S1 Appendix (DOCX)

S1 File (SAV)
