# Supplementary material for: Attitudes of Spanish hospital staff towards COVID-19 vaccination and vaccination rates
Source: PLoS One. 2021 Sep 10;16(9):e0257002. doi: 10.1371/journal.pone.0257002 (PMC8432869; doi:10.1371/journal.pone.0257002)
Supplement: S1 Appendix — (DOCX) [file pone.0257002.s001.docx]

**S1 Appendix. Self-administered questionnaire: COVID-19 vaccination acceptance among hospital workers**

**Q1. Age: Q2. Sex: Q3. Occupation:**

**Q4. How close is your contact with patients?**

1. Very close (daily or almost daily physical contact)
2. Close (no physical contact or physical contact from time to time)
3. I have no contact with patients (exceptional physical contact)

**Q5. Have you received the influenza vaccine during the current season (2020-2021)?**

1. Yes
2. No
3. I don´t remember

**Q6. Did you receive the influenza vaccine during the previous season (2019-2020)?**

1. Yes
2. No
3. I don´t remember

**Q7. Did you receive the influenza vaccine two seasons ago (2018-2019)?**

1. Yes
2. No
3. I don´t remember

**Q8. Is it your intention to receive the COVID-19 vaccine as soon as possible?**

1. Yes, as soon as I can
2. Yes, but I will wait a few weeks/months
3. I don´t know yet what I am going to do
4. I don´t think so
5. Absolutely not

**Q9. Could you please indicate if you belong to any of the following risk groups?**

⃝ Diabetes mellitus

⃝ BMI >40

⃝ Chronic renal disease or nephrotic syndrome

⃝ Cardiovascular disease (including hypertension)

⃝ Neurologic disease

⃝ Respiratory disease

⃝ Hemoglobinopathies and/or anemia

⃝ Hemophilia, other bleeding disorders and chronic bleeding disorders, as well as recipients of blood products and multiple transfusions

⃝ Asplenia or severe splenic dysfunction

⃝ Chronic liver disease (including chronic alcoholism)

⃝ Neuromuscular disease

⃝ Immunosuppression [including primary, secondary viral, drug (also Eculizumab) immunodeficiencies, transplant recipients, and complement deficiency]

⃝ Cancer and malignant blood diseases

⃝ Cochlear implant or pending

⃝ Cerebrospinal fluid fistula

⃝ Celiac disease

⃝ Chronic inflammatory disease

⃝ Prolonged treatment with acetylsalicylic acid

⃝ Pregnancy or puerperium (up to 6 months after delivery)
